# Supplementary material for: Detection of Human Papillomavirus Integration in Brain Metastases from Oropharyngeal Tumors by Targeted Sequencing
Source: Viruses. 2021 Aug 3;13(8):1536. doi: 10.3390/v13081536 (PMC8402651; doi:10.3390/v13081536)
Supplement: Supplementary file 1 [file viruses-13-01536-s001.zip › viruses-1254468-supplementary.pdf]

Supplementary Data

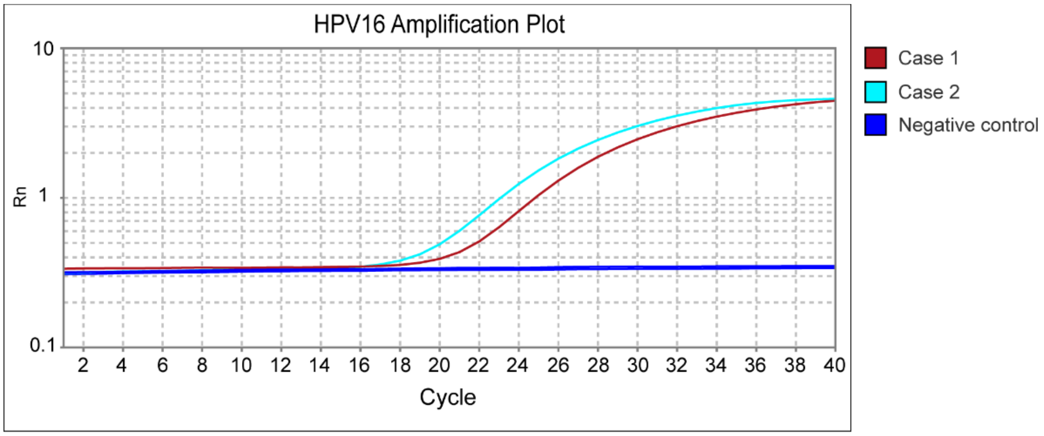

Supplementary Figure 1. PCR assay for HPV16 E6 and HPV18 L1

Total DNA was extracted from metastatic tumors and assayed for the presence of HPV16 and HPV18 DNA using RT-PCR with Taqman probes for HPV16 and HPV18 [24]. PCR of both Case 1 and Case 2 showed amplification of HPV16 DNA, but not HPV18 (data not shown). Negative control was water only.

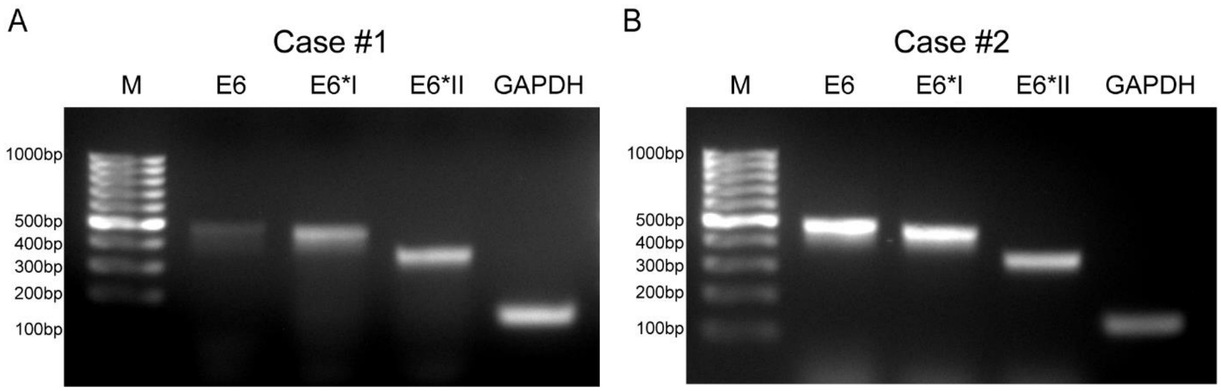

Supplementary Figure 2. PCR identifies specific HPV16 E6 transcripts present in brain metastases

A cDNA library was prepared from total metastatic tumor RNA, followed by PCR using primers specific for three HPV16 E6 splice forms. Forward primers were designed to be specific for full length E6, E6*I, or E6*II, while the reverse primer was located in the HPV16 E7 gene [23]. Both Case 1 and Case 2 expressed the E6*I and E6*II alternate splice forms (a, b). Product sizes are as follows: HPV16 E6-E7 (full length FL) = 499bp, HPV16 E6*I-E7 = 454bp, HPV16 E6*II-E7 = 338bp. M = 100bp DNA ladder (GeneRuler 100bp DNA Ladder, Thermo Fisher, cat. # SM0243). GAPDH primers were from Applied Biosystems (cat. # 4326317E).

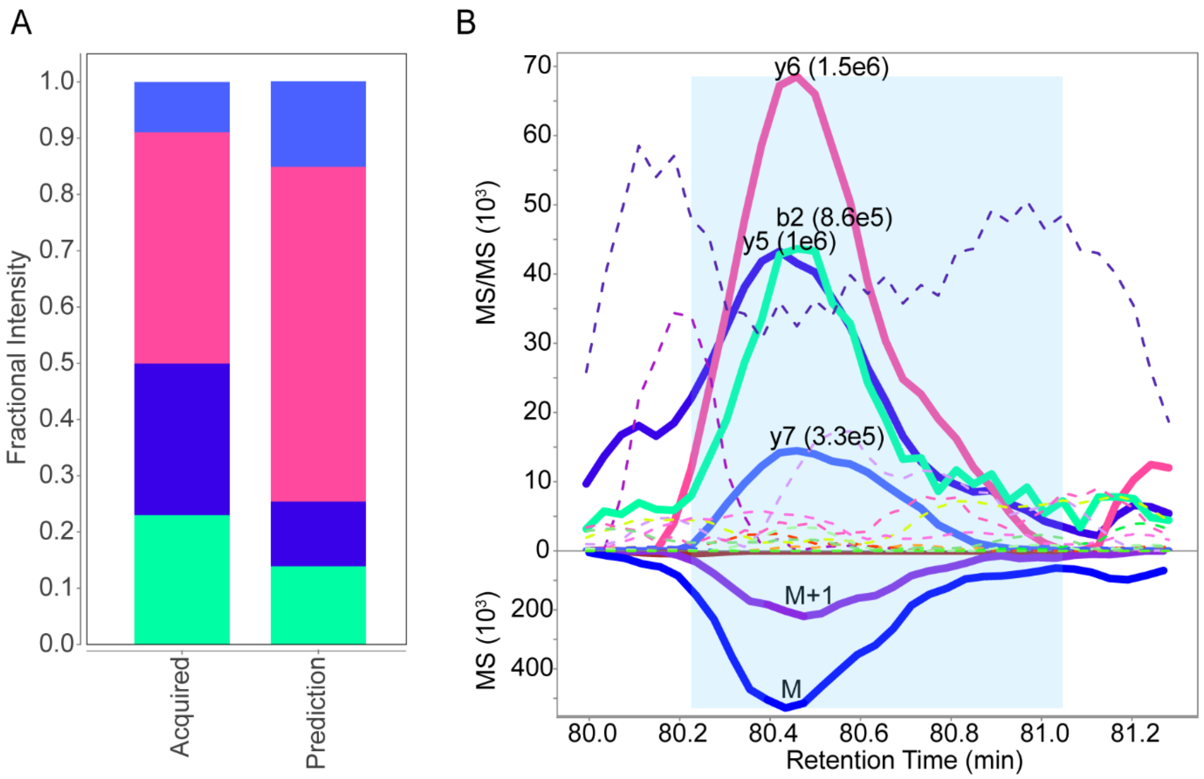

Supplementary Figure 3. Identification of HPV proteins in Case 2 by mass spectrometry

Proteomic analysis of extracted proteins from the Case 2 brain tumor revealed a specific peptide from HPV L1 protein with FLLQAGLK sequence. Correlation of expected and acquired intensity of each MS/MS ion species is represented by different colors. In the butterfly plot (a), these colors correspond to fragment ions in the top chromatograms, while confirmatory precursor ion species are represented by the corresponding bottom chromatograms (b). Solid chromatograms indicate selected diagnostic ions, while dashed chromatograms indicate other ions specific to the peptide sequence.
